# Dim Blue Light at Night Induces Spatial Memory Impairment in Mice by Hippocampal Neuroinflammation and Oxidative Stress

**DOI:** 10.3390/antiox11071218

**Published:** 2022-06-22

**Authors:** Qi Liu, Zixu Wang, Jing Cao, Yulan Dong, Yaoxing Chen

**Affiliations:** Laboratory of Anatomy of Domestic Animals, College of Veterinary Medicine, China Agricultural University, Beijing 100193, China; bs20203050433@cau.edu.cn (Q.L.); zxwang@cau.edu.cn (Z.W.); caojing@cau.edu.cn (J.C.); ylbcdong@cau.edu.cn (Y.D.)

**Keywords:** dim blue light, hippocampus, oxidative stress, neuroinflammation, cognitive plasticity

## Abstract

Light pollution is one of the most serious public problems, especially the night light. However, the effect of dim blue light at night (dLAN-BL) on cognitive function is unclear. In this study, we evaluated the effects of exposure to dLAN-BL in C57BL/6J mice for 4 consecutive weeks. Our results showed dLAN-BL significantly impaired spatial learning and memory and increased plasma corticosterone level in mice. Consistent with these changes, we observed dLAN-BL significantly increased the numbers and activation of microglia and the levels of oxidative stress product MDA in the hippocampus, decreased the levels of antioxidant enzymes Glutathione peroxidase (GSH-Px), Superoxide dismutase (SOD), Gluathione reductase (*Gsr*), total antioxidants (T-AOC) and the number of neurons in the hippocampus, up-regulated the mRNA expression levels of *IL6, TNF-α* and the protein expression levels of iNOS, COX2, TLR4, p-p65, Cleaved-Caspase3 and BAX, and down-regulated the mRNA expression levels of *IL4*, *IL10*, *Psd95*, *Snap25*, *Sirt1*, *Dcx* and the protein expression level of BCL2. In vitro results further showed corticosterone (10uM)-induced BV2 cell activation and up-regulated content of IL6, TNF-α in the cell supernatant and the protein expression levels of iNOS, COX2, p-p65 in BV2 cells. Our findings suggested dLAN-BL up-regulated plasma corticosterone level and hippocampal microglia activation, which in turn caused oxidative stress and neuroinflammation, leading to neuronal loss and synaptic dysfunction, ultimately leading to spatial learning and memory dysfunction in mice.

## 1. Introduction

Since the beginning of life on earth, the cycle of light and dark has been accompanied by the evolution of plants and animals. Based on this cycle, our physiological processes are constantly adapting and changing. In mammals, light detection occurs only in the retina [1], and light detected by the eye is the primary temporal cue for synchronizing circadian rhythms, a process called entrainment [2]. In addition to the rods and cones responsible for imaging functions, there is a third type of photoreceptor cells in the retina that mediate non-imaging functions, namely, intrinsically photosensitive retinal ganglion cells (ipRGCs), which are sensitive to a wavelength of 480 nm [3]. It is well known that the daily light/dark cycle controls nearly all physiological processes, including sleep–wake cycles [4], circadian rhythms [5], learning and memory [6], emotion regulation [7], metabolic function [8] and immune function [9]. The influence of changes in light information, including light cycle, light intensity and light wavelength, on the body’s physiological functions has always been considered and studied.

People are often affected by ambient light pollution in modern life, especially those involved in shift work. Nowadays, lighting at night has become a regular occurrence, and people gradually realize the adverse effects of night-time lighting. Recent studies have shown that dim light at night contributes to the development of many diseases, such as neurodegenerative diseases [10,11], metabolic diseases [12], cardiovascular diseases [13], circadian rhythm disturbances [14], sleep disturbances [15,16]. However, little is known about the effects of dim blue light (~480 nm) at night (dLAN-BL) on physiological functions.

It is well known that external stress can significantly impact memory processes [17,18]. The rodent hippocampus has a well-established role in spatial memory [19]. Previous studies have shown that changes in the external environment may damage the hippocampus, such as noise [20], temperature [21] and smell [22]. Likewise, light is an essential external stressor, and studies have shown that light is a modulator that activates the hippocampus and modulates memory processing [23]. Light has recently been recognized as a modulator able to activate the hippocampus and modulate memory processing. In recent years, many studies have launched discussions on how light affects learning and memory. Fernandez et al. showed that intrinsically photosensitive retinal ganglion cells (ipRGCs) projecting to the suprachiasmatic nucleus (SCN) mediate the effects of light on spatial learning and memory in mice, revealing a direct pathway by which light affects learning and memory [24]. In addition, using the hippocampus as an entry point, many studies have found that irregular light may affect the function of the hippocampus, which in turn affects learning and memory [25,26]. Recent studies have pointed out that dim white light at night (dLAN-WL) can cause changes in hippocampal structure and hippocampal-dependent learning and memory dysfunction [11,13,27]. However, whether dLAN-BL affects hippocampal function is unclear.

Therefore, the present mouse study aimed to investigate the effects of dLAN-BL on (1) spatial learning and memory in mice, (2) the effects on hippocampal structure and (3) the related mechanisms that cause changes in the structure and function of the hippocampus.

## 2. Materials and Methods

### 2.1. Animals and Treatments

Male C57BL6/J mice (*n* = 12, age = 3 weeks old, weight = 18–20 g) were used as model organisms in the current study. The mice were purchased from the Vital River Laboratory Animal Technology Co., Ltd., Beijing, China. All mice were placed in a standard housing environment (23–25 °C, 40–60% humidity, 12h:12h light–dark cycle) and accommodated for one week, with free access to water and food. All experiments were conducted in accordance with the Guide to the Care and Use of Laboratory Animals published by the Animal Welfare Committee of the Agricultural Research Organization, China Agricultural University (Approval No. AW18079102-1-2).

After one week of acclimation, mice were randomly distributed to three groups and were transferred to cabinets kept under a light-dark cycle (12:12, light (~150 lux)/dark (0 lux), Control), a light/dim white light cycle (12:12, light (~150 lux)/dim white light (~5 lux), dLAN-WL) and a light/dim blue light cycle (12:12, light (~150 lux)/dim blue light (~5 lux), dLAN-BL) for 4 consecutive weeks. Daytime lighting was provided with white light emitting diodes (LEDs) fixed on the walls of cabinets, and dim light conditions were created using other white LEDs and blue LEDs kept above the rack on which the mouse cages were placed. The intensity and consistency of lighting were measured using a lux meter. Body weight (BW), food intake and water drinking were monitored regularly for the animals. At the end of the experiments, the behavioral experiments (Y-maze test) were conducted. After the behavioral experiments were over, blood glucose level of the mice was recorded using the blood glucose meter through the tail vein blood, and then, mice were sacrificed for the collection of blood and brain samples.

### 2.2. Y-Maze Test

**Figure 1:**
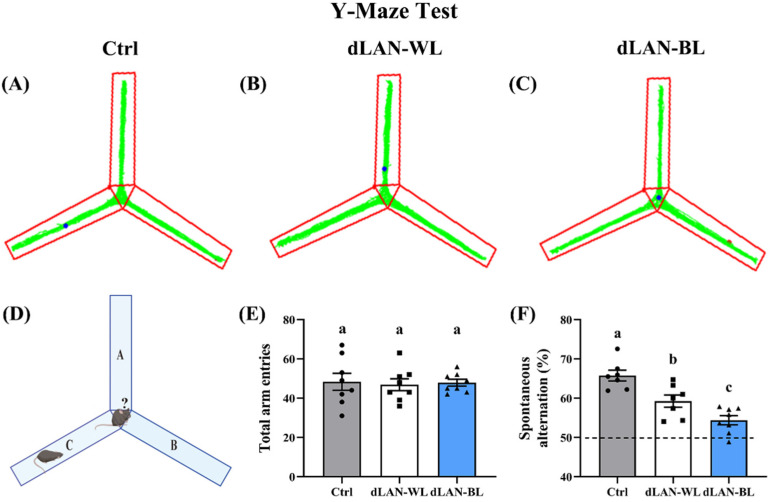
Effect of dim light at night on the ability of spatial learning and memory in mice. (**A**–**C**) Track plot of Y-maze test. (**D**) Schematic diagram of the Y-maze. (**E**) Number of arm entries in the Y-maze test in each group. (**F**) Alternation behavior in the Y-maze test in each group. The data are expressed as the mean ± SEM; *n* = 8 for each group. Values not sharing a common superscript letter (a,b,c) differ significantly at *p* < 0.05; those with the same letter (a,b,c) do not differ significantly (*p* ≥ 0.05). Ctrl: control group, dLAN-WL: dim white light at night group, dLAN-BL: dim blue light at night group.

Y-maze test can be used to detect spontaneous alternation behaviors, which can be used to evaluate mice’s spatial learning and memory abilities. As shown in Figure 1D, the maze has a central area and three opaque arms (labeled A–C, respectively) at an angle of 120° to each other, which are 35 cm long, 5 cm wide and 15 cm high. At the beginning of the experiment, each mouse was placed in the central area and was allowed to move freely in the maze for 8 min. We recorded the total number of arm entries in each group and the order in which they entered each arm, and animal movement was tracked using a computerized tracking system (XR-XY1032; Shanghai Xinsoft Information Technology Co., Ltd., Shanghai, China). Entry into the three different arms in succession was defined as a correct alternative reaction (e.g., A–B–C, A–C–B, B–A–C, B–C–A).

### 2.3. Immunohistochemical Staining

Hippocampus paraffin sections were incubated in rabbit anti-NeuN, rabbit anti-Iba1 and rabbit anti-iNOS primary antibody overnight at 4 °C (1:500, ab177487; 1:500, ab178846; 1:500, ab283655. Abcam, Cambridge, MA, USA). The sections were then rinsed in 0.01 M phosphate buffered solution with Tween-20 (PBST, pH 7.4) and incubated in biotinylated goat anti-rabbit immunoglobulin G (IgG, 1:100, A0277. Beyotime, Shanghai, China) for 2 h at room temperature. After washing, the tissues were incubated in streptavidin-horseradish peroxidase (1:300, A0303. Beyotime, Shanghai, China) for 1.5 h at room temperature. Immunoreactivity was visualized by incubating the tissue sections in 0.01 M phosphate buffered solution (PBS) containing 0.05% 3,3–diaminobenzidine tetrahydrochloride (DAB, Sigma, St. Louis, MO, USA) and 0.003% H_2_O_2_ for 10 min in the dark. The sections were then stained with hematoxylin and mounted. Control slides without the primary antibody were examined in all cases. The localization and distribution of immunoreactive positive materials in the hippocampus were observed using a microscope (BX51, Olympus, Tokyo, Japan).

### 2.4. Sholl Analysis

We acquired the images using an Olympus BX51 microscope (Olympus, Tokyo, Japan) with a 40X objective lens and doubled magnification of individual microglia in the images to demonstrate the morphology of the microglia. For the general analysis of microglia branching, we analyzed the left and right hippocampus of 3 animals per group for a total of 30 random microglia in 3 non-adjacent hippocampal slices per animal for morphological analysis. These microglia were processed separately from the images, and a threshold was applied to convert them from color to binary images, manually adjusting the threshold until only the soma and protrusions of the microglia were visible. We used the Sholl analysis, which counted the number of microglial protrusions intersecting each circle and used it to create a Sholl curve for each cell, which was then averaged to create the average Sholl curve for each animal.

### 2.5. Double Labeling Immunofluorescence

Hippocampus paraffin sections were incubated in rabbit anti-Iba1 primary antibody overnight at 4 °C (1:500, ab178846. Abcam, Cambridge, MA, USA). The sections were then rinsed in 0.01 M PBST (pH 7.4) and incubated in biotinylated goat anti-rabbit IgG (1:300, A0277. Beyotime, Shanghai, China) for 2h at room temperature. After washing, the tissues were incubated in AF-594-streptavidin (1:100, S11227. Sigma, St. Louis, MO, USA) for 1.5 h at room temperature. The sections were then rinsed in 0.01 M PBST (pH 7.4), and 5% goat serum was blocked at room temperature for 30 min, and the paraffin sections were incubated in mouse anti-GR primary antibody overnight at 4 °C (1:100, 66904. Proteintech, Chicago, IL, USA). The sections were then rinsed in 0.01 M PBST (pH 7.4) and incubated in biotinylated goat anti-mouse IgG (1:300, A0286. Beyotime, Shanghai, China) for 2 h at room temperature. After washing, the tissues were incubated in AF-488-streptavidin (1:100, S11223. Sigma, St. Louis, MO, USA) for 1.5 h at room temperature. The localization and distribution of immunoreactive positive materials in the hippocampus were observed using a microscope (BX51, Olympus, Tokyo, Japan).

### 2.6. Real-Time Reverse Transcription-Polymerase Chain Reaction (RT-PCR)

Total RNA was isolated from the hippocampus (*n* = 5) using the TRIzol reagent (CW0580. CWBIO, Beijing, China). First-strand cDNA was synthesized using the RevertAid first-strand cDNA synthesis kit (R312-01. Vazyme, Nanjing, China). RT-PCR amplification was performed using the AceQ qPCR SYBR green master mix (Q111-02. Vazyme, Nanjing, China). Each sample was tested in triplicate, and relative mRNA levels were normalized to the expression levels of the housekeeping gene *Gapdh*. The RT-PCR primers are listed in Table 1.

### 2.7. Western Blot Assay

Hippocampal tissues (*n* = 4) or BV2 cells were lysed in RIPA lysate (CW2333S. CWBIO, Beijing, China) containing 1% protease inhibitor (CW2200S. CWBIO, Beijing, China), and 1% phosphatase inhibitor (CW2383S. CWBIO, Beijing, China) was cracked. Lysates were centrifuged at 14,000× *g* for 15 min at 4 °C, and the supernatant was taken for protein concentration determination. We used a protein assay kit (CW0014. CWBIO, Beijing, China) to quantify protein concentrations. The protein samples were resolved using 8–12% sodium dodecyl sulfate-polyacrylamide gel electrophoresis (SDS-PAGE) and electroblotted onto a polyvinylidene fluoride membrane (Millipore, Billerica, MA, USA). Nitrocellulose membranes were blocked for 60 min using TBST containing 5% fat-free dry milk. They were then incubated in rabbit or mouse primary antibodies (iNOS, 1:1000, ab283655, Abcam; COX2, 1:1000, ab62331, Abcam; TLR4, 1:1000, sc-293072, Santa Cruz Biotechnology; p65, 1:1000, ab16502, Abcam; pp65, 1:1000, 3033S, Cell Signaling Technology) overnight at 4 °C. After washing in TBST, they were incubated in horseradish peroxidase-conjugated goat anti-rabbit IgG (1:8000, CW0103. CWBIO, Beijing, China) or goat anti-mouse IgG (1:8000, CW0102. CWBIO, Beijing, China) for 2 h at room temperature. The protein bands were detected using an enhanced chemiluminescence kit (WBKLS0100. Millipore, Billerica, MA, USA). The protein band intensities were quantified using ImageJ software (4.0.2, Scion Corp., Frederick, MD, USA). The protein level was normalized to the density ratio of β-actin, while the relative protein level in the Ctrl group in vivo or in the control cells in vitro was defined as 100%. Each sample was assayed three times.

### 2.8. Measurements of Antioxidant Activity and Lipid Peroxidation

Hippocampal tissues (*n* = 5) were rapidly homogenized, and clarified lysates were obtained by centrifugation (12,000× *g* for 10 min) at 4 °C. We used a protein assay kit (CW0014. CWBIO, Beijing, China) to quantify protein concentrations, and the tissue extracts were stored at −80 °C for antioxidant activity analysis. Five commercial kits were used to assay the activities of glutathione peroxidase (GSH-Px) (S0056. Beyotime, Beijing, China), superoxide dismutase (SOD) (S0101S. Beyotime, Beijing, China), catalase (CAT) (S0051. Beyotime, Beijing, China), the total antioxidant capability (T-AOC) (S0116. Beyotime, Beijing, China) and the malondialdehyde (MDA) content (S0131S. Beyotime, Beijing, China), according to the manufacturer’s protocol. Each sample was assayed three times.

### 2.9. Cell Culture

BV2 microglia-like cell lines, purchased from the National Infrastructure of Cell Line Resource (NICR), were maintained in Dulbecco’s modified Eagle’s medium supplemented with 10% fetal bovine serum and 4 mM l-glutamine + 100 U/mL penicillin + 100 µg/mL streptomycin + 4500 mg/L Glucose. Cells were grown in a humidified incubator of 95% air/5% CO_2_ at 37 °C. BV2 cells were plated at a density of 8 × 10^3^ cells/well in 96-well plates for MTT experiment, 8 × 10^4^ cells/well in 12-well plates for fluorescent immunocytochemistry analysis and 2 × 10^5^ cells/well in 6-well plates for cytokine experiments and protein expression studies.

### 2.10. MTT Assay

The 3-(4,5-dimethylthiazol-2-yl)-2,5-diphenyltetrazolium bromide (MTT) assay was performed to evaluate the influence of corticosterone (CORT)on the viability of BV-2 cells. Briefly, cells were seeded in a 96-well plate and incubated for 6 h at 37 °C with 5% CO_2_. Next, we replaced the complete medium with the basal medium and continued to culture the cells for 12 h. Then, cells were treated with various concentrations of CORT over a 24 h treatment. Subsequently, MTT solution (5 mg/mL in PBS, Sigma, St. Louis, MO, USA) was added and incubated for 4 h. The supernatant was removed, and the crystals were dissolved with 150 μL of Dimethyl sulfoxide (DMSO, Sigma, St. Louis, MO, USA). The absorbance at 490 nm was detected with a microplate reader (Synergy HT. BioTek, Winooski, VT, USA).

### 2.11. Cellular Immunofluorescence

Cells were plated onto slide glass (801010. Nest, Jiangsu, China). After 1 day of culture, cells were fixed with 95% ethanol. After washing, 5% goat serum was blocked at room temperature for 30 min, and the cells were incubated in mouse anti-GR primary antibody overnight at 4 °C (1:100, 66904. Proteintech, Chicago, IL, USA). The cells were then rinsed in 0.01 M PBST (pH 7.4) and incubated in biotinylated goat anti-mouse IgG (1:300, A0286. Beyotime, Shanghai, China) for 2 h at room temperature. After washing, the tissues were incubated in AF-488-streptavidin (1:100, S11223. Sigma, St. Louis, MO, USA) for 1.5 h at room temperature. The cell slides were taken out of the 12-well plate and placed on a glass slide, mounted with DAPI-containing fluorescent mounting medium (ZLI-9557. ZSGB-BIO, Beijing, China) and stored in the dark. Stained cells were observed using a microscope (BX51, Olympus, Tokyo, Japan).

### 2.12. Enzyme-Linked Immunosorbent Assay (ELISA)

Cell supernatants were collected for the detection of inflammatory factors TNF-α and IL-6 concentrations using ELISA kits according to the manufacturer’s protocol (SLCY. Beijing, China). Absorbance was read at 450 nm with a microplate reader. The intra- and inter-assay variance of the kit was less than 9% and 11%, respectively. All treatments were conducted at least three times.

### 2.13. Statistical Analyses

The data were expressed as the mean ± standard error and analyzed using Graph Pad Prism version 8 (GraphPad Software, La Jolla, CA, USA). Experiments were performed in three independent biological and at least two independent technical replicates. Differences between groups were analyzed using one-way ANOVA followed by Tukey’s multiple comparisons tests. All *p*-values < 0.05 were considered statistically significant.

## 3. Results

### 3.1. Dim Blue Light at Night Impairs Spatial Learning and Memory Function

In order to investigate whether dLAN-BL induced spatial learning and memory impairment, we performed a behavioral analysis—the Y-maze test. As shown in Figure 1E, no significant differences in the total number of arm entries were observed among the groups, which indicated that dim light at night did not exhibit motor dysfunction. By eliminating the interference of motor dysfunction, we evaluated more accurately the effect of dLAN-WL and dLAN-BL on spatial learning and memory impairment. In the test, compared with the control group, the percentage of alternate behaviors of mice in the dLAN-WL group and dLAN-BL group decreased by 9.89% (*p* = 0.0037) and 17.29% (*p* < 0.0001) (Figure 1F), respectively. It is worth noting that the percentage of alternate behavior of mice in the dLAN-BL group was 8.21% (*p* = 0.0192) lower than that in the dLAN-WL group (Figure 1F). Figure 1A–C showed the track plot of mice in the Y-maze test. The above results indicated that dLAN-WL caused spatial learning and memory impairment in mice, while dLAN-BL caused more severe spatial learning and memory impairment.

### 3.2. Dim Blue Light at Night Induces Loss of Hippocampal Neurons

**Figure 2:**
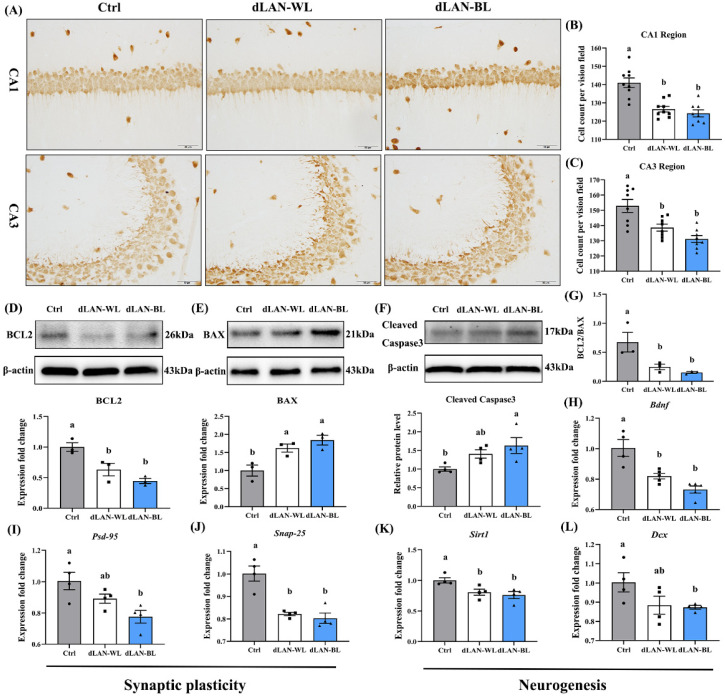
Effect of dim light at night on hippocampal neurons in mice. (**A**) NeuN immunohistochemical staining in the CA1 and CA3 regions of the hippocampus in the different groups. Bar =50 μm. (**B**) The NeuN-positive cells in the hippocampal CA1 region (*n* = 9). (**C**) The NeuN-positive cells in the hippocampal CA3 region (*n* = 9). (**D**) Relative protein levels of BCL2 in the hippocampus (*n* = 3). (**E**) Relative protein levels of BAX in the hippocampus (*n* = 3). (**F**) Relative protein levels of cleaved-caspase3 in the hippocampus (*n* = 3). (**G**) The ratio of BCL-2 and BAX protein expression levels (*n* = 3). (**H**) The mRNA levels of Bdnf (*n* = 4). (**I**) The mRNA levels of Psd95 (*n* = 4). (**J**) The mRNA levels of Snap25 (*n* = 4). (**K**) The mRNA levels of Sirt1 (*n* = 4). (**L**) The mRNA levels of Dcx (*n* = 4). Differences were assessed using one-way ANOVA. The result represents the mean ± standard error of the mean. Values not sharing a common superscript letter (a,b) differ significantly at *p* < 0.05; those with the same letter (a,b) do not differ significantly (*p* ≥ 0.05). Ctrl: control group, dLAN-WL: dim white light at night group, dLAN-BL: dim blue light at night group.

Spatial learning and memory impairment are closely related to hippocampal structure damage. In order to examine the effect of dim light at night on the density of neurons in the hippocampus, we used immunohistochemistry to detect the expression of NeuN in the hippocampal CA1 and CA3 regions of mice in each group, as shown in Figure 2A. The results showed that the density of neurons in the hippocampal CA1 and CA3 areas in the dLAN-WL group was 10.42% (*p* < 0.0001) and 10.19% (*p* = 0.0014) lower than in the control group (Figure 2B,C), respectively, while the density of neurons in the hippocampal CA1 and CA3 areas in the dLAN-BL group was 12.23% (*p* < 0.0001) and 15.12% (*p* < 0.0001) lower than in the control group (Figure 2B,C), respectively. The above results indicated that dLAN-WL and dLAN-BL both induced loss of hippocampal neurons. These findings substantiated the damage of dim light at night on the hippocampal structure.

To investigate whether dim light at night induced apoptosis, we detected the protein levels of Cleaved-Caspase3, BCL2 and BAX in the hippocampus. The results showed that compared with the control group, the protein levels of BCL2 in the dLAN-WL group and the dLAN-BL group were decreased by 36.80% (*p* = 0.0139) and 55.40% (*p* = 0.0021), respectively (Figure 2D). However, the protein levels of BAX in the dLAN-WL group and the dLAN-BL group were increased by 62.33% (*p* = 0.0166) and 84.25% (*p* = 0.0043), respectively (Figure 2E). Compared with the control group, dLAN-WL and dLAN-BL induced a decrease in the BCL2/BAX ratio (Figure 2G), which altered the balance between the anti-apoptotic protein BCL2 and the pro-apoptotic protein BAX. Activation of Caspase3 is the most critical apoptotic executive event in apoptosis, and dLAN-BL also induced significant changes in Cleaved-Caspase3 protein levels in the hippocampus. In the dLAN-BL group, the expression level of Cleaved-Caspase3 was significantly 63.08% higher than in the control group (*p* = 0.0123) (Figure 2F). The above results indicated that dim light at night, especially dLAN-BL, induced apoptosis in the hippocampus.

### 3.3. Dim Blue Light at Night Impairs Hippocampal Synaptic Function and Neurogenesis

To investigate the effect of dLAN-BL on hippocampal synaptic function and neurogenesis, we detected the mRNA expression levels of Bdnf, Psd95, Snap25, Sirt1 and Dcx. Compared with the control group, the expressions of synaptic function and neurogenesis-related molecules in the dLAN-WL group were reduced by 18.42% (Bdnf, *p* = 0.0024), 11.27% (Psd95, *p* > 0.05), 17.96% (Snap25, *p* = 0.0005), 11.88% (Dcx, *p* > 0.05) and 11.88% (Sirt1, *p* > 0.05) (Figure 2H–L), while the expressions of synaptic function and neurogenesis-related molecules in the dLAN-BL group were reduced by 27.18% (Bdnf, *p* = 0.0001), 22.79% (Psd95, *p* = 0.0046), 19.85% (Snap25, *p* = 0.0002), 13.00% (Dcx, *p* = 0.0473) and 13.00% (Sirt1, *p* = 0.0473) (Figure 2H–L). These results suggested that dim light at night, especially dLAN-BL, impaired hippocampal synaptic function and neurogenesis in the hippocampus.

### 3.4. Dim Blue Light at Night Induces Oxidative Stress in the Hippocampus

**Figure 3:**
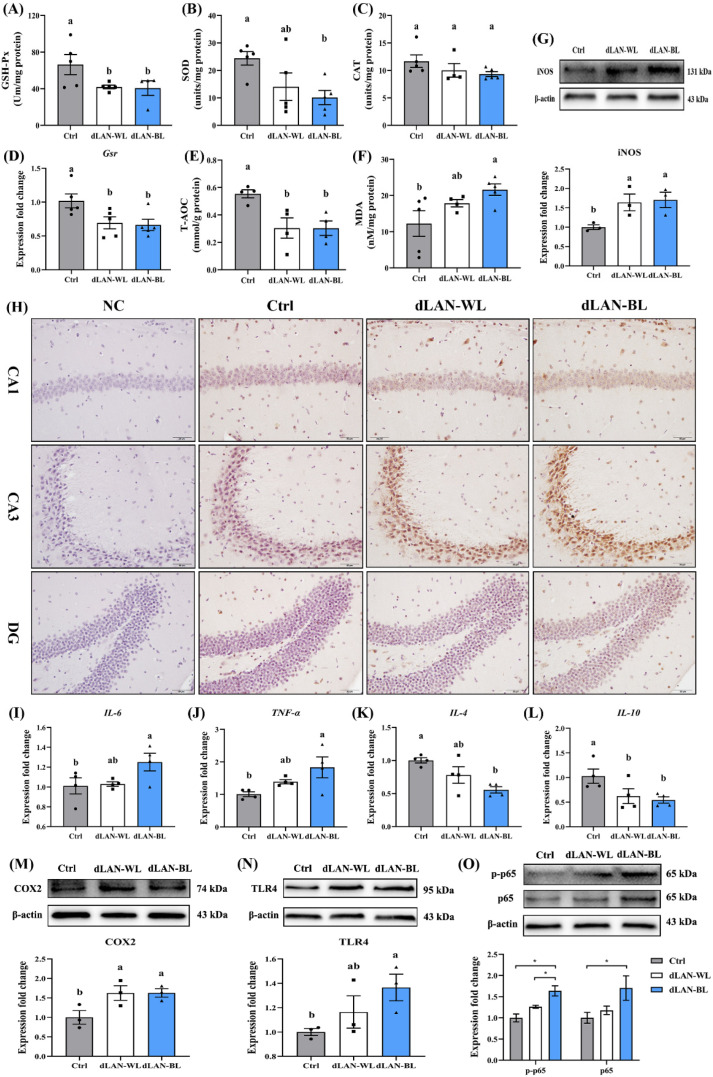
Effects of dim blue light at night on oxidative stress and inflammation in the hippocampus. (**A**) The levels of GSH-Px in the hippocampus (*n* = 5). (**B**) The levels of SOD in the hippocampus (*n* = 5). (**C**) The levels of CAT in the hippocampus (*n* = 5). (**D**) The mRNA levels of Gsr in the hippocampus (*n* = 5). (**E**) The levels of T-AOC in the hippocampus (n = 5). (**F**) The levels of MDA in the hippocampus (*n* = 5). (**G**) Relative levels of iNOS in the hippocampus (*n* = 3). (**H**) Images of the immunohistochemical iNOS in the different experimental groups. Bar =50 μm. (**I**) The mRNA levels of cytokines IL-6 in the hippocampus (*n* = 4). (**J**) The mRNA levels of cytokines TNF-α in the hippocampus (*n* = 4). (**K**) The mRNA levels of cytokines IL-4 in the hippocampus (*n* = 4). (**L**) The mRNA levels of cytokines IL-10 in the hippocampus (*n* = 4). (**M**) Relative levels of COX2 in the hippocampus (*n* = 3). (**N**) Relative levels of TLR4 in the hippocampus (*n* = 3). (**O**) Relative levels of p-p65 and p65 in the hippocampus (*n* = 3). Differences were assessed using one-way ANOVA. The result represents the mean ± standard error of the mean. Values not sharing a common superscript letter (a,b) or marking (*) differ significantly at *p* < 0.05; those with the same letter (a,b) do not differ significantly (*p* ≥ 0.05). Ctrl: control group, dLAN-WL: dim white light at night group, dLAN-BL: dim blue light at night group.

To investigate whether dim light at night induced oxidative stress in the hippocampus, we examined six antioxidant parameters, including antioxidant enzymes (GSH-Px, SOD, CAT and Gsr), total antioxidant capacity (T-AOC) and malondialdehyde (MDA). Compared with the control group, the expression of antioxidant enzymes and the level of total antioxidant capacity in the hippocampus of the mice in the dLAN-WL group were reduced by 36.91% (GSH-Px, *p* = 0.0441), 42.36% (SOD, *p* > 0.05), 14.19% (CAT, *p* > 0.05), 31.90% (Gsr, *p* = 0.0273) and 45.05% (T-AOC, *p* = 0.0109) (Figure 3A–E), while the expression of antioxidant enzymes and the level of total antioxidant capacity in the hippocampus of the mice in the dLAN-BL group were reduced by 38.68% (GSH-Px, *p* = 0.0463), 58.50% (SOD, *p* = 0.0148), 20.04%(CAT, *p* > 0.05), 34.94% (Gsr, *p* = 0.0175) and 45.20% (T-AOC, *p* = 0.0107) (Figure 3A–E). In contrast, we observed a significant increase in MDA levels, the end product of lipid peroxidation, in the hippocampus of the dLAN-BL group. Compared with the control group, the MDA content was significantly increased by 77.81% (*p* = 0.0176) in the hippocampus of the dLAN-BL group (Figure 3F). These results suggested that dim light at night, especially dLAN-BL, induced oxidative stress in the hippocampus.

### 3.5. Dim Blue Light at Night Induces Inflammation in the Hippocampus

Inducible nitric oxide synthase (iNOS) is involved in nitric oxide production and is often associated with neuroinflammation in the brain. We examined the expression of iNOS in the hippocampus of different groups. The results showed that the expressions of iNOS in the hippocampus in the dLAN-WL and the dLAN-BL groups were 63.86% (*p* = 0.0407) and 70.39% (*p* = 0.0286) higher than those in the control group (Figure 3G), respectively. More importantly, we localized it by immunohistochemistry and found that the above changes mainly occurred in the CA1 and CA3 region of the hippocampus (Figure 3H). These results indicated that dLAN-WL and dLAN-BL both increased the expression level of iNOS in the hippocampus and had a specific impact on the occurrence of hippocampal inflammation.

Activated microglia produce pro-inflammatory cytokines, such as IL-6 and TNF-α, and IL4 and IL10 are important anti-inflammatory cytokines that play a crucial role in suppressing pathological inflammatory processes. ANOVA revealed significant effects of dLAN-BL on the mRNA levels of cytokines IL6, TNF-α, IL-4 and IL-10 in the hippocampus. We found that the levels of pro-inflammatory factors IL-6 and TNF-α in the hippocampus of the dLAN-BL group were significantly increased by 23.74% (*p* = 0.0402) and 81.82% (*p* = 0.0147) compared with the control group (Figure 3I,J), while the anti-inflammatory factor IL-4 and IL-10 levels were significantly reduced by 41.48% (*p* = 0.0036) and 47.21% (*p* = 0.0234) (Figure 3K–L). In addition, dLAN-BL also significantly increased hippocampal pro-inflammatory factor COX2 by 62.89% (*p* = 0.0325) (Figure 3M).

Consistent with these changes, dLAN-BL also activated the NF-κB pathway in the hippocampus. We observed that the protein levels of hippocampal TLR4 in the dLAN-BL group were significantly higher than those in the control group by 36.56% (*p* = 0.0424) (Figure 3N). Additionally, we examined the protein levels of p-p65 and p65 in the NF-κB pathway. The results showed that the protein levels of p-p65 and p65 in the hippocampus of the dLAN-BL group were 63.75% (*p* = 0.0025) and 70.52% (*p* = 0.040) higher than those of the control group (Figure 3O), respectively, which indicated that dLAN-BL induced the activation of the NF-κB pathway. The above results indicated that dLAN-BL promoted inflammation, adversely affecting the hippocampus.

### 3.6. Dim Blue Light at Night Induces Activation of Hippocampal Microglia

**Figure 4:**
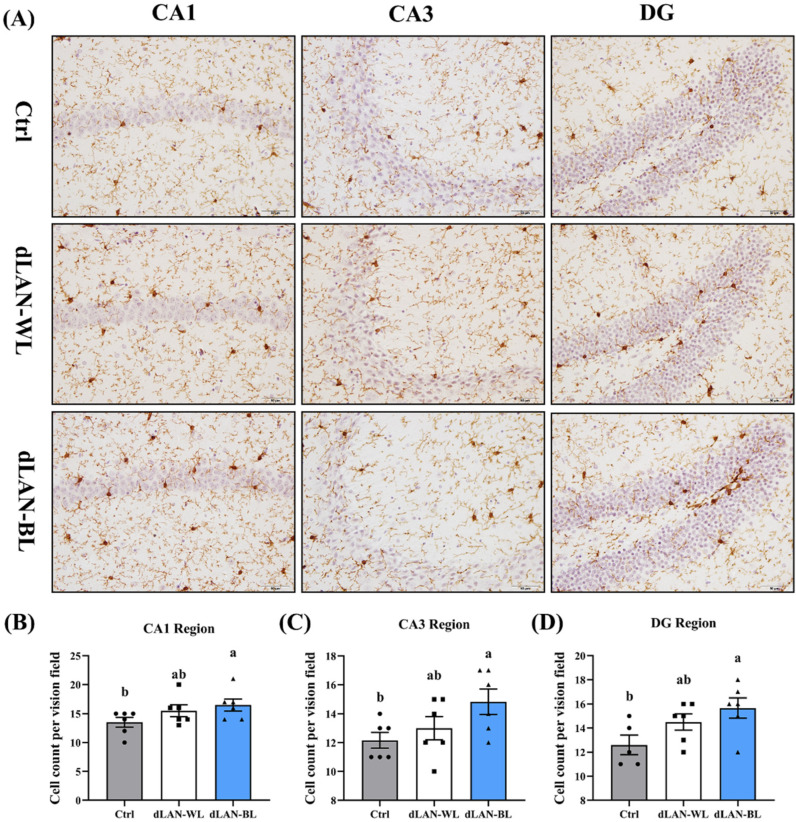
Effects of dLAN on the number of microglia in the CA1, CA3 and DG regions of the hippocampus. (**A**) Images of the immunohistochemical microglia in the different groups. The immunohistochemical results were processed using Image J. Bar =50 μm. (**B**) Number of microglia in the hippocampal CA1 region (*n* = 6). (**C**) Number of microglia in the hippocampal CA3 region (*n* = 6). (**D**) Number of microglia in the hippocampal DG region (*n* = 6). Differences were assessed using one-way ANOVA. The result represents the mean ± standard error of the mean. Values not sharing a common superscript letter (a,b) differ significantly at *p* < 0.05; those with the same letter (a,b) do not differ significantly (*p* ≥ 0.05). Ctrl: control group, dLAN-WL: dim white light at night group, dLAN-BL: dim blue light at night group.

To further examine whether dim light at night affected neuroinflammation, we first examined the number of microglia in the hippocampus of different groups (Figure 4A). The results showed that the numbers of microglia in the hippocampal CA1, CA3 and DG regions in the dLAN-WL and the dLAN-BL groups were 14.81% (*p* > 0.05) and 22.22% (*p* = 0.0470), 6.85% (*p* > 0.05) and 21.92% (*p* = 0.0251), 15.08% (*p* > 0.05) and 24.34% (*p* = 0.0161) higher than those in the control group (Figure 4B–D), respectively. These results suggested that dLAN-BL induced an inflammatory response in the mouse hippocampus.

**Figure 5:**
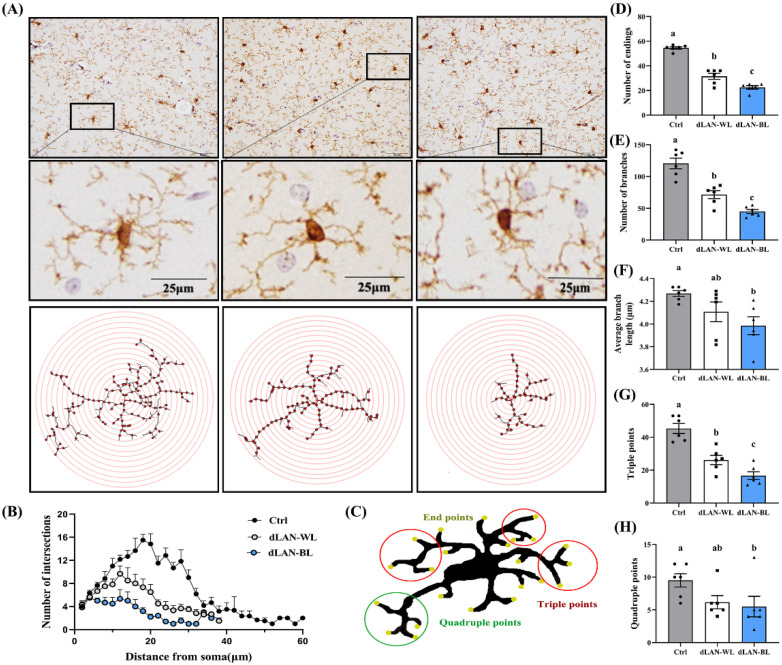
Effects of dLAN on microglia morphology in the hippocampus. (**A**) Schematic diagram of Sholl analysis. The results of Sholl analysis were processed using ImageJ. Bar = 25 μm. (**B**) Quantification of microglial ramification complexity using Sholl analysis (*n* = 6). (**C**) Schematic example of a skeletonized microglia showing endpoints, triple junctions (junctions with exactly three branches) and quadruple junctions (junctions with exactly four branches). (**D**) Analysis of the microglial endpoints (*n* = 6). (**E**) Analysis of the microglial branches (*n* = 6). (**F**) Analysis of the average branch length of microglia (*n* = 6). (**G**) Analysis of the triple points of microglia (*n* = 6). (**H**) Analysis of the quadruple points of microglia (*n* = 6). Differences were assessed using one-way ANOVA. The result represents the mean ± standard error of the mean. Values not sharing a common superscript letter (a,b) differ significantly at *p* < 0.05; those with the same letter (a,b) do not differ significantly (*p* ≥ 0.05). Ctrl: control group, dLAN-WL: dim white light at night group, dLAN-BL: dim blue light at night group.

Microglia activation in vivo is accompanied by a morphological change in which microglia in an activated state transitions from a branched to an amoeba morphology. To further assess the function of microglia, we also analyzed the morphology of microglia in the hippocampus, including the number of endpoints, number of branch points, average branch length, the triple junction (junction with exactly three branches) and quadruple junctions (junctions with exactly four branches) and used the Sholl analysis to reflect the complexity of microglia. Figure 5A is an illustration of the Sholl analysis. Compared with the control group, the number of the endpoints, branch numbers and triple junctions of hippocampal microglia in the dLAN-WL group were significantly reduced by 42.20% (*p* < 0.0001), 40.75% (*p* < 0.0001) and 42.28% (*p* = 0.0002), respectively (Figure 5D–G). However, compared with the control group, the number of endpoints, branch numbers, average branch lengths, triple junctions and quadruple junctions in the dLAN-BL group were significantly reduced by 58.72% (*p* < 0.0001), 62.71% (*p* < 0.0001), 6.64% (*p* = 0.0108), 63.24% (*p* < 0.0001) and 42.11% (*p* = 0.0361) (Figure 5D–H). Similar results were obtained from the Sholl analysis (Figure 5B). It is worth noting that compared with the dLAN-WL group, the number of endpoints, branches and triple junctions of the hippocampal microglia in the dLAN-BL group was reduced by 28.57% (*p* = 0.0023), 37.06% (*p*= 0.0091) and 36.31% (*p* = 0.0275) (Figure 5D–E,G). These results suggested that dLAN-WL induced partial changes in hippocampal microglia morphogenesis, and dLAN-BL exacerbated this effect.

### 3.7. Corticosterone Induces Activation of BV2 Cells

To explore the mechanism by which dim light at night induced hippocampal damage, we detected the levels of corticosterone in mouse plasma. Compared with the control group, the level of corticosterone in the plasma of mice in the dLAN-WL and the dLAN-BL groups was significantly increased by 5.7% (*p* = 0.0052) and 4.8% (*p* = 0.0197), respectively.

**Figure 6:**
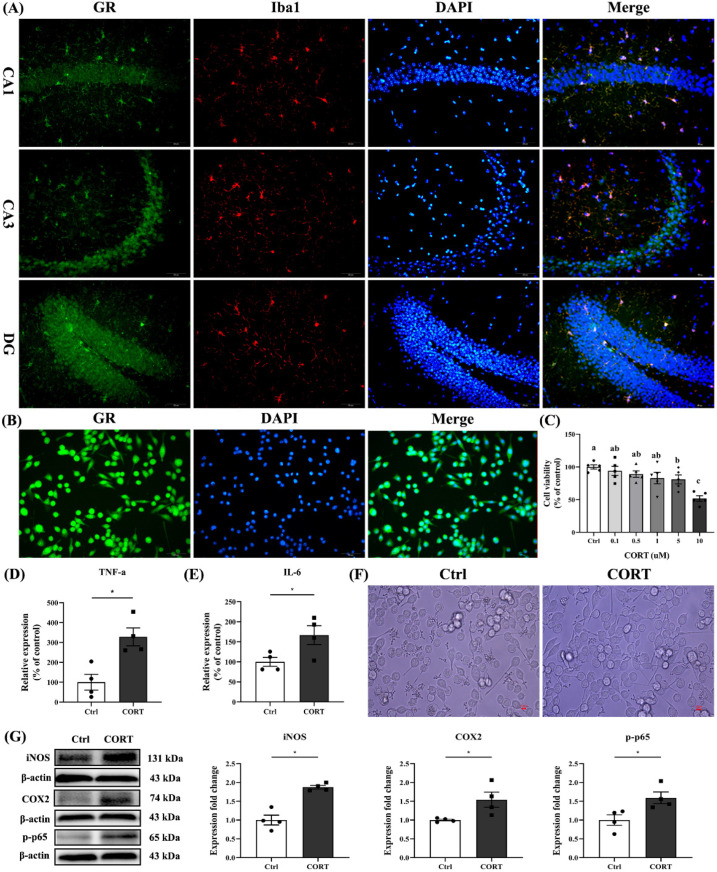
Corticosterone-induced activation of microglia. (**A**) Immunofluorescence of GR in the hippocampus of mice. Iba1 marked microglia; GR marked glucocorticoid receptor; DAPI marked nuclei, respectively. Bar = 50 µm. (**B**) Immunofluorescence of GR in the BV2 cells. Bar = 50 µm. (**C**) Determination of the effect of CORT in BV-2 cells using MTT assay. BV-2 cell treated with CORT with indicated concentrations (*n* = 6). (**D**) TNF-α detected in the supernatant of BV-2 cells (*n* = 4). (**E**) IL-6 detected in the supernatant of BV-2 cells (*n* = 4). (**F**) Effects of CORT intervention on microglia morphology. (**G**) Protein expression levels of iNOS, COX2 and p-p65 detected by immunoblotting in BV-2 cells (*n* = 4). Differences were assessed using t-tests. The result represents the mean ± standard error of the mean. Values not sharing a common superscript letter (a,b,c) or marking (*) differ significantly at *p* < 0.05; those with the same letter (a,b,c) do not differ significantly (*p* ≥ 0.05). Ctrl: control group, CORT: BV-2 cells treated with CORT (10 μM) group.

However, there was no significant difference in plasma corticosterone levels between dLAN-WL and dLAN-BL mice (*p* = 0.6107) (Results not shown). Double immunofluorescence labeling showed that glucocorticoid receptors were expressed on microglia in various regions of the hippocampus (Figure 6A). Furthermore, the results of cellular immunofluorescence indicated that BV2 cells expressed glucocorticoid receptors (Figure 6B). To further validate these findings, we treated BV2 cells with CORT to study the effect of CORT on microglia activation. The MTT results indicated that corticosterone damaged microglia in a dose-dependent manner (Figure 6C). The data of the ELISA assay showed that the contents of TNF-α and IL-6 in the supernatant of BV2 cells treated with CORT (10 μM) were 228.13% (*p* = 0.0091) and 66.61% (*p* = 0.0430) higher than those of the control group, respectively (Figure 6D–E). CORT pre-exposure induced significantly less branched morphology in BV2 cells overall (Figure 6F). In addition, the protein expression levels of iNOS, COX2 and p-p65 in CORT-treated cells were 87.32% (*p* = 0.0008), 54.15% (*p* = 0.0375) and 59.01% (*p* = 0.0303) higher than those in the control group. These results suggested that elevated corticosterone levels induced microglia activation and led to inflammation.

## 4. Discussion

Light has a profound influence on the physical health of mammals, including humans. In addition to illuminating the environment for vision (i.e., image forming), light also regulates non-image-forming processes [28]. In recent years, there has been increasing evidence that exposure to abnormal light, especially at night, can damage the body’s health. In the present study, we found dLAN-BL induced elevated plasma corticosterone levels and neurotoxicity, providing new insights into how ambient light guides human health.

The Y-maze is a classic behavioral method that can be used to examine spatial learning and memory in mice [29]. In this study, we found no significant difference in the total number of arm accesses between groups of mice over 8 min, suggesting neither dLAN-WL nor dLAN-BL impaired the mice’s motor abilities. Meanwhile, we found dLAN-WL significantly reduced the percentage of spontaneous alternating behaviors in mice. Importantly, dLAN-BL caused more severe spatial learning and memory impairment. Similar to our study, the continuous exposure of mice to dLAN-WL impaired their performance in the Morris water maze test and the Barnes maze test [11,30]. However, this is the first study to investigate the effects of dLAN-BL on spatial learning and memory in mice. The above results suggested dLAN-WL impaired spatial learning and memory in mice, and dLAN-BL exacerbated this impairment.

The hippocampus is the center of mice’s spatial learning and memory function, and spatial learning and memory impairments have been reported to be closely related to hippocampal structural damage and synaptic dysfunction [31]. In this study, using NeuN as a marker, we found dLAN-WL and dLAN-BL induced a decrease in the number of neurons in the CA1 and CA3 regions of the hippocampus. Consistent with the previous study, disruption, increased pyknosis and chromatolysis (characteristics of damaged neurons) were observed in the hippocampal and cerebral cortex neurons of the dim light at night exposed mice [11]. Apoptosis is an important pathway for neuronal loss [32]. In the present study, an elevated expression of BAX and reduced expression of BCL2 were found in the hippocampus of dLAN-WL and dLAN-BL mice, and an elevated expression of Cleaved-Caspase3 was found in the hippocampus of dLAN-BL mice, indicating dim light at night, especially dLAN-BL, induced apoptosis in the hippocampus.

“Neurogenesis” and “Synaptogenesis”, which are closely related to learning and memory functions, are the two main pathways involved in brain development. Synapses are where information is transmitted between neurons, and reduced synaptic function is associated with impaired cognitive performance. BDNF is expressed in the hippocampus and plays an important role in synaptic plasticity, neuronal survival and memory formation by binding and activating its affinity receptor TrκB. Dim light at night has been reported to reduce hippocampal BDNF expression levels [33]. We first observed a decrease in the mRNA level of Bdnf induced by dLAN-BL. In addition, correlation analysis found the mRNA level of Bdnf was significantly positively correlated with the percentage of spontaneous alternation behavior in the Y-maze (Result not shown). Moreover, as an upstream molecule of BDNF, SIRT1 has multiple roles in mammalian brain development, and Sirt1 deletion in the mouse brain leads to reduced performance in cognitive tasks (spatial learning and memory) and neurogenesis [34]. Subsequently, we analyzed the mRNA expression levels of Psd95, Snap25, Sirt1 and Dcx in the hippocampus, which indicated that dim light at night, especially dLAN-BL, impairs synaptic function and neurogenesis in the mouse hippocampus. The above results indicated that dLAN-BL induced neuronal apoptosis, resulting in neuronal loss and synaptic dysfunction.

Microglia are resident macrophages in the central nervous system (CNS) with immune surveillance [35]. Chronic microglial activation is a hallmark of many neuropathologies, and activated microglia perpetuate inflammation by releasing pro-inflammatory and neurotoxic factors that ultimately exacerbate neurotoxicity and neurodegeneration [36]. Ionized Ca^2+^-binding adapter protein 1 (Iba1) is a conserved, intracellular and Ca^2+^-binding adapter protein of pro-inflammatory nature, which is selectively expressed by microglia and macrophages and has long been utilized as a microglial marker [37]. We found dLAN-BL increased the number of Iba1-positive cells in the mouse hippocampal CA1, CA3 and DG regions, indicating dLAN-BL induced neuroinflammation in the hippocampus. Similar to our study, dLAN-WL increased the number of Iba1-positive cells in the mouse hypothalamus [38], suggesting a link between night-time light and neuroinflammation. In addition, the morphology of microglia is highly plastic and closely related to its functional biological state [39], and the definition of microglial activation was initially primarily based on changes in its morphology [40]. In this study, we examined several metrics reflecting microglia morphology, including the number of endpoints, number of branch points, average branch length, triple junctions (junctions with exactly three branches), quadruple junctions (junctions with exactly four branches), and the complexity of microglia was analyzed using the Sholl analysis. We found dLAN-BL induced a reduction in microglia’s average branch length and branch complexity, suggesting dLAN-BL induced activation of hippocampal microglia. These results suggested dLAN-BL induced an activation of hippocampal microglia in mice.

Notably, microglia have three phenotypes: M0, M1 and M2 [35]. Under normal circumstances, microglia are in the M0 type. Activated microglia are classified into M1 and M2 types based on the expression of specific cell surface molecules and specific cytokines [41]. M1 microglia can activate iNOS, activate the NF-κB pathway and produce pro-inflammatory cytokines, such as TNF-α, IL-1β and IL-6. In contrast, M2-type microglia can release anti-inflammatory factors and exert anti-inflammatory effects [42]. Nitric oxide (NO) is one of the most essential neural signaling molecules mediating hippocampal learning and memory functions [43]. Inducible nitric oxide synthase (iNOS) is involved in nitric oxide production and is often associated with neuroinflammation in the brain [44]. It was reported the photoperiod affects the diurnal expression of iNOS, which may in turn influence hippocampal function [45]. In this study, we found both dLAN-WL and dLAN-BL induced a significant increase in iNOS expression levels. Notably, we found this change mainly occurred in the CA1 and CA3 regions of the hippocampus but not in the DG region. As far as we know, this was first reported by us. In addition, dim light at night has been reported to induce the release of pro-inflammatory factors and the reduction in anti-inflammatory factors and to induce activation of the NF-κB pathway [13,46]. Similar to previous studies, our current findings suggested dim light at night caused increases in IL6 and TNF-α levels and decreases in IL4 and IL10 levels. Furthermore, the levels of TLR4 and pp65 were significantly increased in the dLAN-BL group. Taken together, the results suggested that dim light at night, especially dLAN-BL, induced neuroinflammation in the hippocampus.

The brain is known to consume a lot of oxygen to function properly and is considered a factory of free radicals/reactive oxygen species, which are particularly susceptible to oxidative stress [47]. The brain has a higher demand for oxygen and therefore consumes 20% more oxygen than the rest of the body [48]. Oxidative stress is one of the predisposing factors leading to brain tissue damage and neurodegenerative diseases. Antioxidant enzyme (e.g., SOD, catalase) activity is decreased in the brain of AD patients. Dim light at night has been reported to cause oxidative stress [33]. In order to explore the cause of hippocampal neuron damage, we first analyzed the antioxidant levels in the hippocampus of mice exposed to dim light at night and found that light at night, especially dLAN-BL, caused antioxidant enzymes (except Catalase) in the hippocampus, and decreased levels reduce the total antioxidant capacity of the hippocampus, which in turn causes elevated levels of the only oxidative stress product, malondialdehyde. Similar results have been reported in other studies [11], which suggested dim light at night, especially dLAN-BL, caused oxidative stress in the hippocampus and resulted in neuronal damage in the hippocampus.

Interestingly, how does dim light at night affect hippocampal function? Studies have shown that as a circadian-rhythm-related hormone, corticosterone is susceptible to light exposure [49]. Previous studies have shown that long-term exposure to constant light (CCL) caused an increase in plasma corticosterone levels and decreased hippocampal glucocorticoid receptor (GR) phosphorylation in mice [50]. The rhythm of CORT is known to be unchanged in mice exposed to dim light at night [51]. Another study in diurnal squirrels noted that continuous darkness increased corticosterone levels, whereas no such changes were found in nocturnal mice [52], suggesting the CORT response to dim light at night exposure also exhibits interspecies differences. There is limited information on how dim light at night affects corticosterone. In the present study, we observed both dLAN-WL and dLAN-BL induced significant increases in plasma corticosterone levels in mice. However, unlike acute stress, dLAN-WL and dLAN-BL did not induce dramatic changes in plasma corticosterone in mice. We speculated that prolonged chronic light stimulation may result in a modest increase in corticosterone levels. Next, we further explored the effect of corticosterone on microglial activation. Multiple steroid hormone receptors, including GR and MR, have been reported in microglia [53]. Studies have shown that CORT inhibited BV2 microglia proliferation with cytotoxic effects, an effect involving GR but not MR [54]. In this study, the co-localization results of GR and Iba1 showed GR was expressed on microglia in the hippocampal CA1, CA3 and DG regions, and in vitro experiments also proved that GR was expressed on BV2 cells. We found that corticosterone (10uM) promoted the release of pro-inflammatory factors and the expression of pro-inflammatory proteins in BV2 cells, suggesting CORT induced the activation of microglia.

Disruption of circadian rhythms by environmental conditions can lead to changes in body homeostasis, from behavior to body metabolism. The circadian system maintains internal 24 h behavioral and physiological fluctuations whose inconsistency with the external light–dark (LD) cycle can lead to negative health outcomes [52], of which the light–dark cycle is the most reliable environment factor [55]. Melatonin and corticosterone, the two most recognized circadian output hormones, exhibit marked circadian rhythm [56]. Studies have shown that melatonin synthesis is highly sensitive to light. Dim night-time light has been reported to significantly reduce nocturnal melatonin levels in rats [57]. Unlike rats, melatonin levels in mice vary widely, and many strains, including the C57BL/6J mice used in this study, do not even produce the hormone. In this study, we focused on the role of corticosterone. However, little is known about how dim light at night affects corticosterone and the mediating role of corticosterone in dim light at night impairing cognitive function. This is a shortcoming of our study.

## 5. Conclusions

In summary, this study provided the first evidence that dim light at night, especially dLAN-BL, leads to impaired spatial learning and memory in mice. With the above changes, neuroinflammation and oxidative stress occurred in the hippocampus, and hippocampal neurons underwent apoptosis, eventually leading to neuronal loss and impaired synaptic function, which may be related to the increase in plasma corticosterone. Our findings suggested dim light at night, especially dLAN-BL, leads to neurotoxicity and cognitive-behavioral deficits. This is the first study to report the effects of dLAN-BL on the learning and memory function in mice, providing new insights into how ambient light guides human health.

## Figures and Tables

**Table 1:** Primers for real-time PCR.

Gene Name	Primer Sequence	Product Size	Accession No.
*IL-6*	F: TAGTCCTTCCTACCCCAATTTCC	76	NC_000071.7
	R: TTGGTCCTTAGCCACTCCTTC		
*TNF-α*	F: CCCTCACACTCAGATCATCTTCT	61	NC_000083.7
	R: GCTACGACGTGGGCTACAG		
*IL-4*	F: GGTCTCAACCCCCAGCTAGT	102	NC_000077.7
	R: GCCGATGATCTCTCTCAAGTGAT		
*IL-10*	F: AGCCTTATCGGAAATGATCCAGT	229	NC_000067.7
	R: GGCCTTGTAGACACCTTGGT		
*Bdnf*	F: TTACCTGGATGCCGCAAACAT	101	NC_000068.8
	R: TGACCCACTCGCTAATACTGTC		
*Sirt1*	F: GCTGACGACTTCGACGACG	101	NC_000076.7
	R: TCGGTCAACAGGAGGTTGTCT		
*Psd-95*	F: TCCGGGAGGTGACCCATTC	83	NC_000077.7
	R: TTTCCGGCGCATGACGTAG		
*Snap-25*	F: CAACTGGAACGCATTGAGGAA	177	NC_000068.8
	R: GGCCACTACTCCATCCTGATTAT		
*Gapdh*	F: CCGAGAATGGGAAGCTTGTC	232	NC_000072.7
	R: TTCTCGTGGTTCACACCCATC		

## Data Availability

Data are contained within the article.
